# Combined perfusion and metabolic imaging of small cell lung cancer with dual-isotope LAFOV-PET

**DOI:** 10.1007/s00259-025-07355-3

**Published:** 2025-05-27

**Authors:** Nasir Gözlügöl, Hasan Sari, Tereza Losmanová, Ali Afshar-Oromieh, Axel Rominger, Federico Caobelli

**Affiliations:** 1 Department of Nuclear Medicine, Inselspital, Bern University Hospital, University of Bern, Freiburgstrasse 18, Bern, 3010 Switzerland; 2 Institute of Tissue Medicine and Pathology, University of Bern, Bern, Switzerland

A 70-year-old female patient with previous smoking history and no prior history of pulmonary lesions underwent a ^82^Rb-Chloride positron emission tomography/computed tomography (PET/CT) scan for the evaluation of myocardial ischemia due to atypical anginal symptoms. Images were acquired on a long-axial-field-of-view (LAFOV) PET/CT scanner (Biograph Vision Quadra, Siemens Healthineers, Knoxville, TN, USA). While no myocardial perfusion abnormalities were detected, an incidental hyperperfused pulmonary lesion was observed in the right perihilar region in rest conditions, previously unknown and not documented in prior imaging (transaxial fused image, yellow arrow, A) (Fig. 1). A subsequent [^18^F]FDG PET/CT was performed 7 days later on the same scanner and showed intense uptake (SUVmax 16.6, SUVmean 4.6), (transaxial fused image, red arrow, B).Time-activity-curves were generated on dynamic ^82^Rb PET/CT using the PKIN module of the PMOD software (PMOD Technologies LLC, Zurich, Switzerland), which enabled quantitative assessment of blood flow based on a one-tissue compartment model. Blood flow within the lesion appeared to be slightly higher after administration of stress agent (Regadenoson 400 µg, C). This incidental finding significantly altered the patient’s clinical management. The lesion was confirmed as small cell lung carcinoma (SCLC) by biopsy, which showed sparse infiltrates of SCLC cells in cytology specimens, characterized by crush artifacts, necrosis, neuroendocrine marker expression (and a high proliferation rate (Ki67 > 80%, D). The characteristic cytomorphology of SCLC, including small, densely packed tumor cells with hyperchromatic nuclei, scant cytoplasm, and a high nuclear-to-cytoplasmic ratio was also observed (Haematoxylin-Eosin, E).

**Fig. 1:**
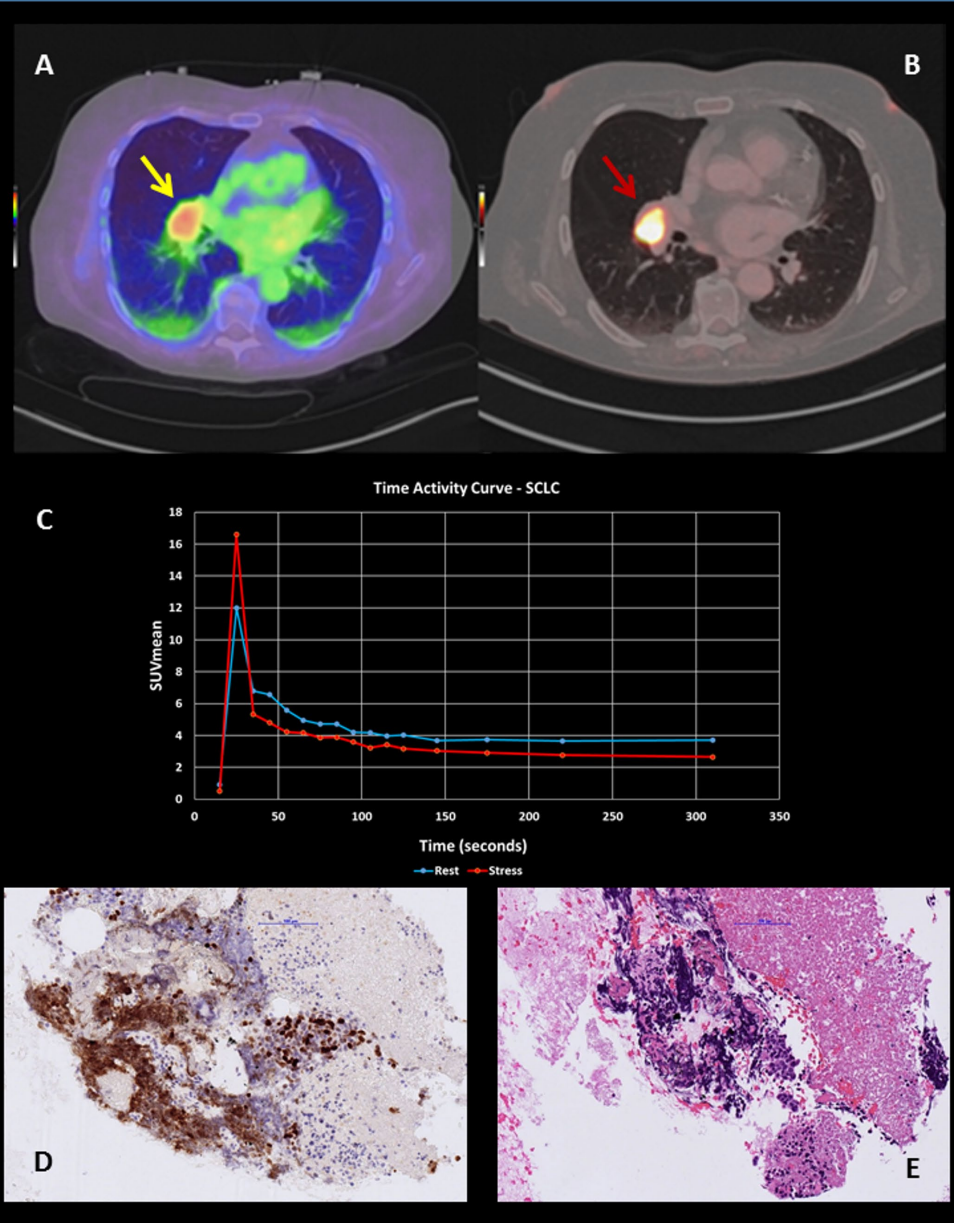
Incidental finding of a hyperperfused right perihilar lung lesion on [^82^Rb]Rb PET/CT performed for myocardial perfusion assessment (**A**). The lesion demonstrated intense FDG uptake on follow-up [^18^F]FDG PET/CT (**B**). Dynamic [^82^Rb]Rb PET-derived time-activity curves (**C**) revealed elevated perfusion under Regadenoson-induced stress. Cytology confirmed small cell lung carcinoma (SCLC) with high proliferative index (Ki-67 >80%) and typical morphology (**D**, **E**)

Absolute flow quantification within tumoral lesions is expected to be feasible on LAFOV PET, wherein dynamic, total body imaging can allow to generate parametric mapping in the organs of interest. In fact, the dynamic, whole-body data acquisition enables the assessment of myocardial perfusion while also extracting perfusion information from incidental lesions throughout the body, providing a unique opportunity for comprehensive oncologic and metabolic evaluation in a single scan [1, 2].

This case highlights the potential of ^82^Rb-Chloride PET/CT imaging with a LAFOV scanner to incidentally detect and characterize extracardiac findings. To note, ^82^Rb is not validated for the assessment of the perfusion within the tumor. However, quantification of ^82^Rb PET was validated against microspheres, an approach that is also suitable for the perfusion analysis in tumor lesions. It remains to be determined what the normal reference values are and whether variations in blood flow may reflect differences in tumor aggressiveness [3]. Incorporating perfusion information derived from ^82^Rb PET into [^18^F]FDG PET-based metabolic assessment of a suspected lung lesion might be the key to enhance characterization of benign or malignant lung lesions [4]. In dedifferentiated, highly aggressive tumors, angiogenesis is more abundant compared to well-differentiated tumors. However, this tumor-induced neovascularization is typically disorganized, immature, and inefficient, leading to regional hypoperfusion and hypoxia despite the presence of numerous vessels [5–7]. The resulting imbalance between oxygen supply and metabolic demand induces a metabolic shift towards aerobic glycolysis, known as the “Warburg effect,” characterized by high glucose consumption and intense [^18^F-] FDG uptake [8–11]. Furthermore, regions of severe hypoxia often correlate with necrotic tumor areas, reflecting insufficient perfusion within highly proliferative tumors [12]. This perfusion-metabolism mismatch in PET imaging may therefore serve as a marker of tumor aggressiveness and vascular inefficiency [13, 14]. It should be noted that ^82^Rb is readily available, allows robust quantification, and benefits from a short half-life facilitating imaging directly before FDG administration. However, some weaknesses should be considered, such as lack of formal validation and potential challenges in assessing small lesions due the tracer’s high positron range, resulting in deteriorated spatial resolution [15, 16].

In our case, the incidental detection of SCLC during a cardiac ^82^Rb PET scan prompted rapid histological confirmation and oncologic evaluation. Without considering extracardiac ^82^Rb uptakes, the lesion might have remained undetected at this early stage, as it was not visible on prior imaging. The integrated perfusion and metabolic information contributed to the high suspicion of malignancy, providing greater diagnostic confidence and eventually leading to initiation of oncologic treatment. This case highlights the potential role of LAFOV PET in the early, non-invasive characterization of unexpected findings.

## Electronic supplementary material

Below is the link to the electronic supplementary material.

Supplementary Material 1 (56.2 KB)

## Data Availability

The datasets generated during the current study are available from the corresponding author on reasonable request.
