# Supplementary material for: Combined perfusion and metabolic imaging of small cell lung cancer with dual-isotope LAFOV-PET
Source: Eur J Nucl Med Mol Imaging. 2025 May 27;53(3):1359–62. doi: 10.1007/s00259-025-07355-3 (PMC12860840; doi:10.1007/s00259-025-07355-3)
Supplement: Supplementary file 1 — Supplementary Material 1 (56.2 KB) [file 259_2025_7355_MOESM1_ESM.pptx]

## Slide 1
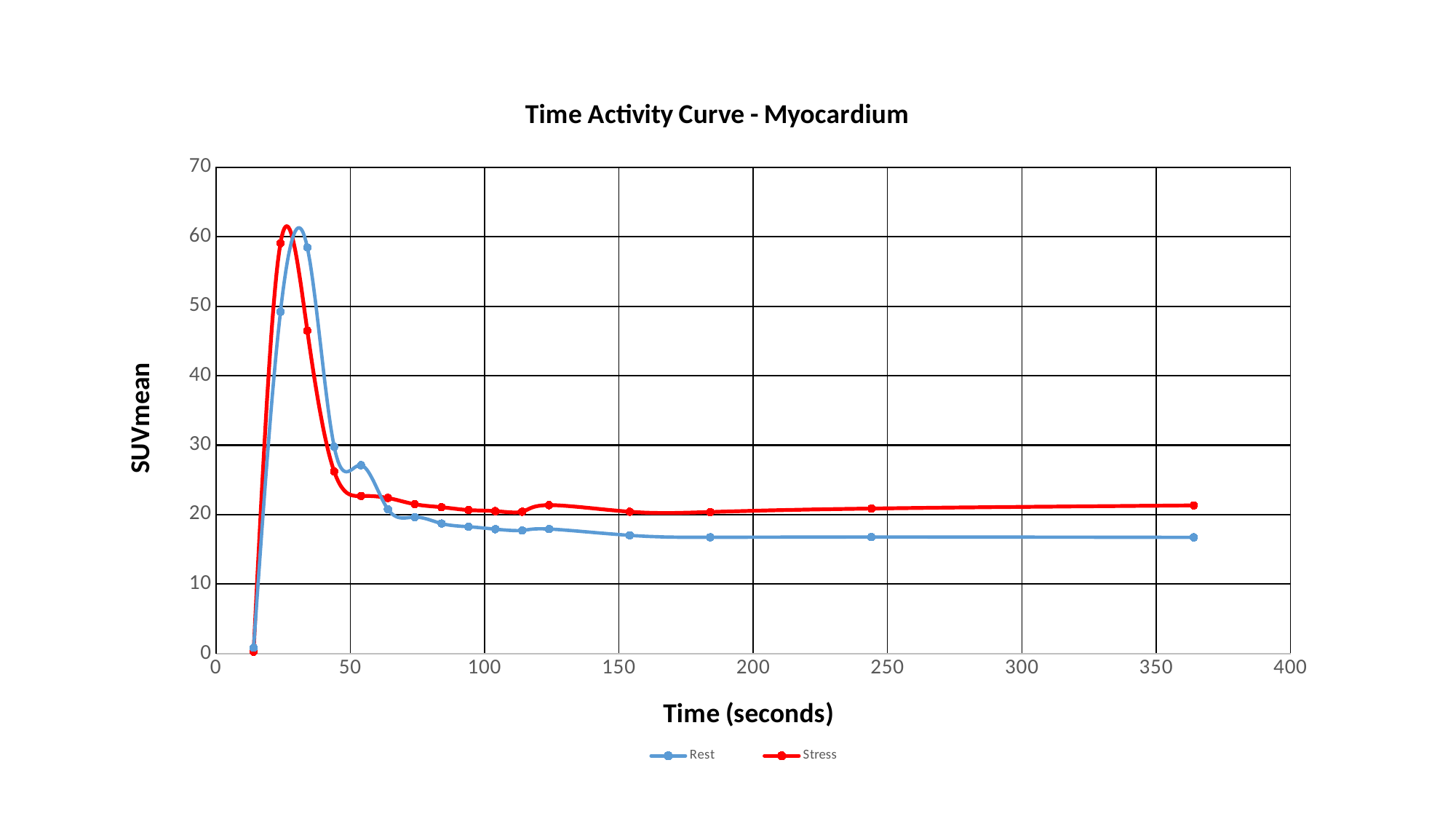

### Chart: Time Activity Curve - Myocardium
| Category | | |
|---|---|---|

## Slide 2
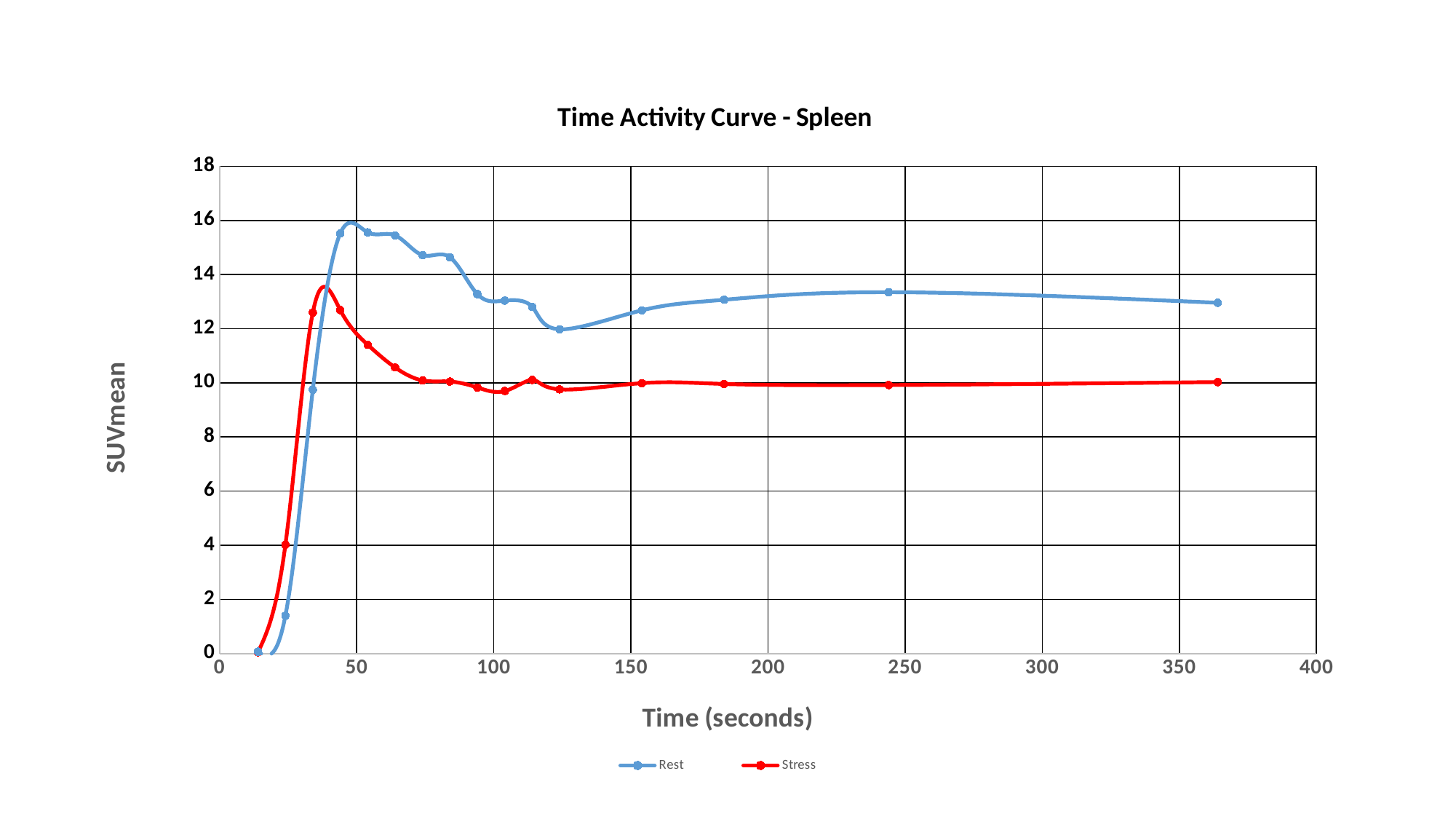

### Chart: Time Activity Curve - Spleen
| Category | | |
|---|---|---|

## Slide 3
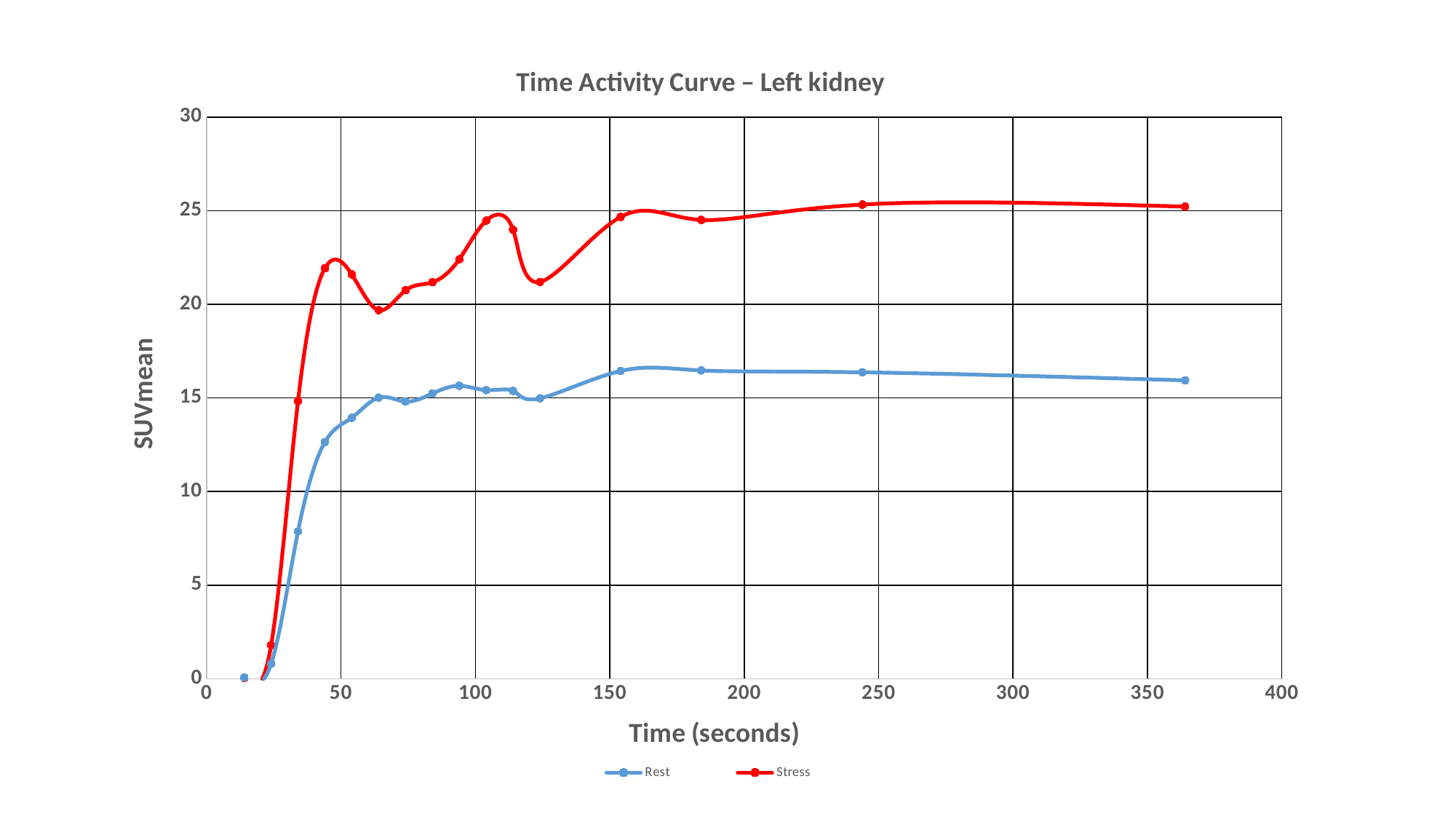

### Chart: Time Activity Curve – Left kidney
| Category | | |
|---|---|---|

## Slide 4
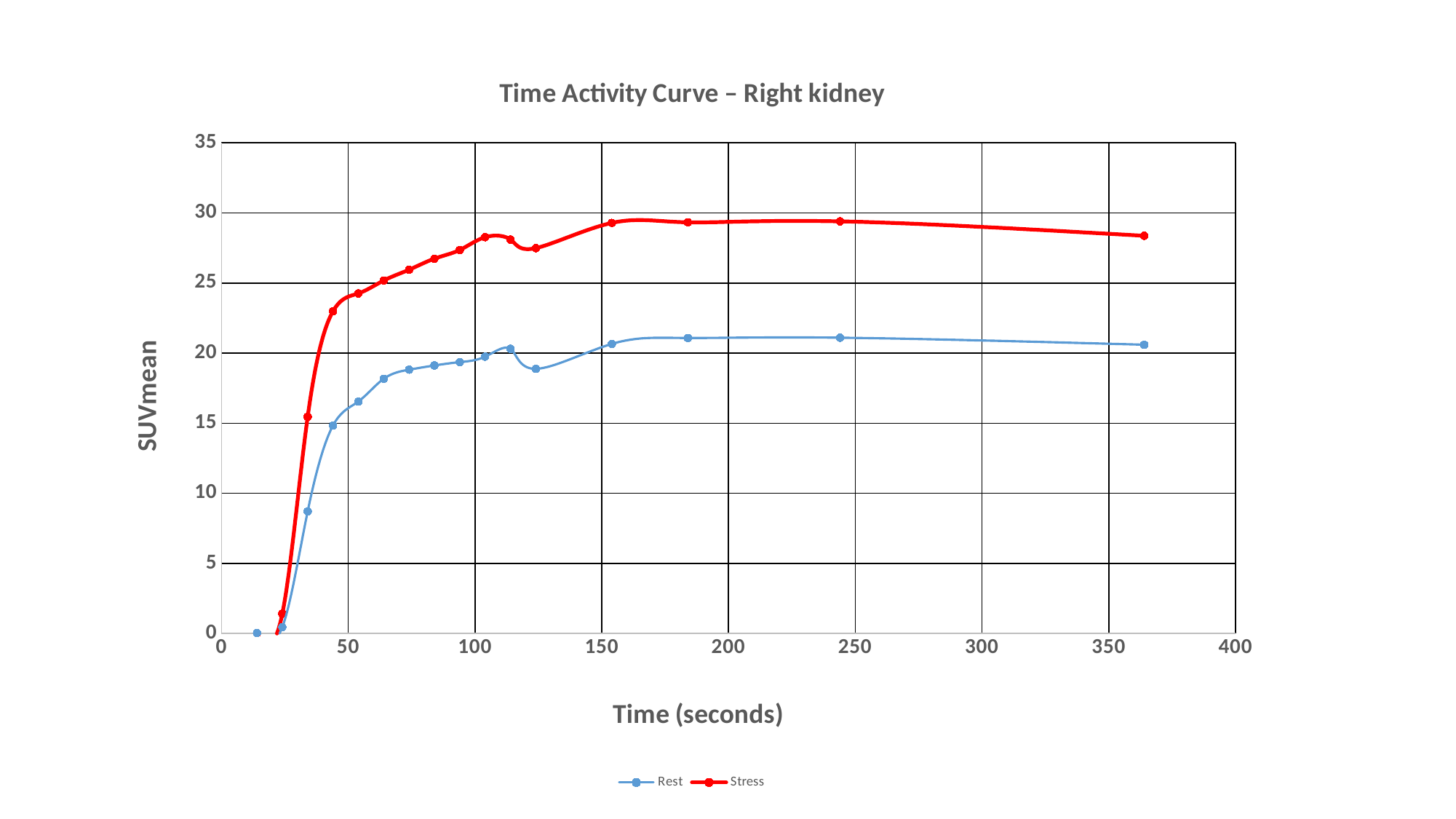

### Chart: Time Activity Curve – Right kidney
| Category | | |
|---|---|---|
